# The metaverse in education: Definition, framework, features, potential applications, challenges, and future research topics

**DOI:** 10.3389/fpsyg.2022.1016300

**Published:** 2022-10-11

**Authors:** Xinli Zhang, Yuchen Chen, Lailin Hu, Youmei Wang

**Affiliations:** College of Education, Wenzhou University, Wenzhou, China

**Keywords:** metaverse, metaverse in education, metaverse for learning, virtual reality, augmented reality, extended reality

## Abstract

The declaration of the COVID-19 pandemic forced humanity to rethink how we teach and learn. The metaverse, a 3D digital space mixed with the real world and the virtual world, has been heralded as a trend of future education with great potential. However, as an emerging item, rarely did the existing study discuss the metaverse from the perspective of education. In this paper, we first introduce the visions of the metaverse, including its origin, definitions, and shared features. Then, the metaverse in education is clearly defined, and a detailed framework of the metaverse in education is proposed, along with in-depth discussions of its features. In addition, four potential applications of the metaverse in education are described with reasons and cases: blended learning, language learning, competence-based education, and inclusive education. Moreover, challenges of the metaverse for educational purposes are also presented. Finally, a range of research topics related to the metaverse in education is proposed for future studies. We hope that, *via* this research paper, researchers with both computer science and educational technology backgrounds could have a clear vision of the metaverse in education and provide a stepping stone for future studies. We also expect more researchers interested in this topic can commence their studies inspired by this paper.

## Introduction

With the COVID-19 pandemic declared in 2020, humanity was forced to live in a society being non-face-to-face with each other (Koo, 2021; Kye et al., 2021; Kim et al., 2022; Lee et al., 2022). In particular, a range of activities in the physical world has transited into the virtual world. Telecommuting, online meetings, distance learning, online shopping, etc., have become a natural part of human life. As a result, the human need to expand the boundaries of the physical world has been accelerated, triggering the yearning for a more advanced virtual world (Suzuki et al., 2020). Owing to the breakthrough of VR (virtual reality), AR (augmented reality), AI (artificial intelligence), blockchain, etc., the metaverse, a 3D digital space with collapsed virtual and real boundaries, has provoked increasing attention. It has been recognized as the next generation of the Internet (Hwang and Chien, 2022), which is about to dramatically change how we interact with the world.

2021 was known as the first year of the metaverse (Zhang et al., 2022a). As global metaverse research is flourishing, the metaverse has been touted as a future education trend, with great potential (Choi and Kim, 2017; Dwivedi et al., 2022; Gartner, 2022; Guo and Gao, 2022; Hwang and Chien, 2022; Park and Jeong, 2022; Park and Kim, 2022; Rospigliosi, 2022; Shin, 2022; Tlili et al., 2022). The presence of the metaverse usually couples with multiple new technologies (Kang, 2021). However, the previous literature rarely discussed the metaverse from the perspective of education but focused much on the metaverse-related technologies in education separately. As an emerging item, the majority of educational researchers might be unaware of what the metaverse is, its components, and its application in the educational field. Therefore, this research paper aims to review several representative articles to give a clear view of the metaverse in education, including its definition, framework, typical features, potential applications, challenges, and future research issues. The main contributions of this paper include the following points:

- The origin, definition, and typical features of the metaverse are discussed with the perspectives taken from state-of-the-art studies.
- A detailed framework of the metaverse in education is proposed, along with the discussion about features of metaverse-based learning compared with in-person classroom learning and screen-based remote learning.
- Potential applications, challenges, and future research topics of the metaverse in education are presented.

The remainder of this paper is arranged as follows: In section 2, we introduce the visions of the metaverse, including its origin, definitions, and shared features. In section 3, discussions are provided to define “the metaverse in education” and a framework of the metaverse in education is proposed; moreover, the features of metaverse-based learning are compared with in-person classroom learning and screen-based remote learning. In section 4, potential applications of the metaverse in education are described with reasons and cases. Section 5 mainly discusses the challenges of the metaverse for educational purposes. Finally, a variety of research topics related to the metaverse in education for future studies is proposed in Section 6. We hope that, *via* this paper, researchers with both computer science and educational technology backgrounds could have a clear picture of the metaverse in education and provide a stepping stone for future studies.

## Origin, definition, and features of the metaverse

### Origin of the metaverse

Metaverse is a compound word combined with “meta-” (beyond; transcending) and “verse” (the root of “universe,” cosmos; the whole world), which denotes a new virtual universe created beyond the real world. The term “metaverse” was first coined in the 1992 cyberpunk science fiction novel Snow Crash written by American novelist Neal Stephenson (Stephenson, 1992; Joshua, 2017). In this novel, humans could freely access a 3D space that reflects the real world through digital agents (avatars) and interact with each other. Over the next three decades, the metaverse concept was more vividly depicted in science fiction movies, such as Ready Player One, Lucy, and The Matrix (Zhao et al., 2022). At that time, the metaverse envisioned by films, could not come into being in reality. In this decade, the rapid progress of emerging technologies, such as wearable devices and three-dimensional (3D) photography, has helped people to get access to the virtual world. In March 2021, The sandbox game Roblox was listed in New York under the halo of “the first stock of the metaverse”; in October, Facebook proclaimed its rebrand scheme and changed its name to “Meta.” Since then, extensive efforts have gradually been carried out by countries across the world to make it a reality. This sleeping “lion” was truly awakened.

### Definition and features of the metaverse

As a new term, researchers discussed the metaverse with broad insights. In 2007, the Acceleration Studies Foundation, a metaverse research organization, took the first step to put forward the metaverse roadmap and propose the metaverse is a fusion of both virtually-enhanced physical reality and physical-persisted virtual space (Smart et al., 2007; Kye et al., 2021). In light of the two axes: ‘augmentation versus simulation’ and ‘intimate versus external’, four scenarios were categorized in the metaverse roadmap: augmented reality, lifelogging, virtual worlds, and mirror worlds. This was the early idea of the metaverse under limited technology. After that, differing metaverse descriptions have been reported with the advancement of virtual technologies. Mark Zuckerberg unveiled his schemes to build Facebook a “metaverse”: an embodied online world where people can present themself, work, play, and socialize with avatars, often in the form of headsets or glasses (Bobrowski, 2021; Zuckerberg, 2021). Similarly, Roblox founder David Baszucki (Jeon, 2021) defined the metaverse as a place that combines high-fidelity communication with a new way to tell stories borrowing from mobile gaming and the entertainment industry. Center for Journalism Studies of Tsinghua University (2021) reported that the metaverse is a created internet application and social form fused with the virtual and real world; it is shaped by integrating many types of new technologies: XR (extended reality) to provide a real and immersive experience, digital twins to map the real world, blockchain to construct credit system, economic system, and exchange system, etc.; it realizes the close connection of the social, economic, and identity systems in the virtual and real world, and allows the user to content production and edit in the metaverse. In this case, the definition of the metaverse seems not to reach a consensus, and there is no single, unified entity called the metaverse; rather, some applications conceive the metaverse with some of its possible features.

In the early 2000s, multiuser role-play game communities such as Second Life (released in 2003) and World of Warcraft (launched in 2004) began to draw the attention of millions. They were what are now being called antecedents of the metaverse; however, they were not hit the bull’s eye in the years since (Wiederhold, 2022). In recent years, several online games or social networks continuing that trend have regained widespread popularity. The hottest mentioned one is Roblox, a sandbox game under the user-generated content (UGC) mode where users can create their own virtual world and enjoy real-time interaction with other players. At the same time, assets can be generated through game development and item sales to other players. As mentioned by David Baszucki (Jeon, 2021), there are eight fundamentals of the metaverse: identity, social, immersive, low fiction, variety, anywhere, economy, and civility. In addition, ZEPETO is the most representative social network application from South Korea, with close to 200 million worldwide users in 2020 (NAVER Z Corp, 2022). In ZEPETO, everyone can customize their unique avatars through selfies and dress-up, and use their virtual identities to interact with others remotely through body movements, voice calls, and taking snapshots; not only that, users are allowed to generate revenue by making and selling AR fashion items. It is reported that the world-famous female Kpop group Blackpink’s virtual fan signing event has been held at ZEPETO with surpassed 30 million users’ participation, and the avatar performance has exceeded 40 million views. Scholars indicated such digital games are prototypes of the metaverse with typical features (Hwang and Chien, 2022; Prieto et al., 2022). In this respect, the metaverse is more than so-called digital games or social networks in a conventional meaning, and some peculiarities need to be taken into account. Accordingly, the shared features of the metaverse can be synthesized and summarized as follows:

#### Collection of technologies

As the studies reported (Center for Journalism Studies of Tsinghua University, 2021; Kang, 2021; Shen et al., 2021; Sparkes, 2021; Lv et al., 2022; Park and Kim, 2022; Prieto et al., 2022), the metaverse is not merely a new entity for VR or AR, but a collection of a set of emergent technologies like 5G, AI, VR, AR, digital twins, blockchain, holography, or IoT (Internet of Things). The technological framework might be built for specific realms such as entertainment, commerce, education, etc., and their components and functions can be different according to the needs.

#### Convergence of the virtual and real world

This is the basic feature of the metaverse. As the metaverse roadmap stated, the metaverse is a fusion of virtually-enhanced reality and physical-persisted virtual space (Smart et al., 2007; Kye et al., 2021), that is, the metaverse includes both the items mapped or augmented from the real world and the creations produced in the virtual world. The gap between the virtual and the physical world will be narrowed or even eliminated in the metaverse, which enables the user’s experience in the metaverse more immersive, multi-sensory, and close to authentic.

#### Rapid and free access

With the support of high-speed networks such as 5G/6G, users can use smart wearable devices (e.g., headsets or glasses) to enter the metaverse world instantly without being constrained by either time or location (Ayiter, 2019; Prieto et al., 2022). From this point of view, it realizes free and rapid access for users by switching between the real world and the virtual world remotely and seamlessly.

#### Digital identity

Instead of a static image, in the metaverse, each user could customize his/her unique digital identity in the form of an avatar (Davis et al., 2009; Dionisio et al., 2013; Park and Kim, 2022). The construction of the digital identity is more user-defined and more advanced than before, for instance, it could edit the details of the avatar’s face (Wei et al., 2004), body (Kocur et al., 2020), and even facial expression (Murphy, 2017). It is the surrogate identity of users in the virtual world which reflects the user’s persona and represents the ego in the real world. In addition, avatars can be manipulated and controlled by users with the help of real-time tracking technologies (Saragih et al., 2011; Genay et al., 2021). In this case, the living 3D representation of users (i.e., digital identity) plays an important role in ownership, interactivity, embodiment, and socialization in the metaverse world.

#### Immersive and multisensory experience

In the metaverse, the vivid and colorful virtual scenes modeled by technologies can deliver users a deep feeling of immersion (Shin, 2022; Zhao et al., 2022). With the aid of technologies such as sensors, VR, AR, or IoT, users are allowed to interact with the created virtual items or the items projected from the real world through moving, manipulating, or clicking, thereby greatly motivating users’ multiple senses (Koo, 2021; Jovanović and Milosavljević, 2022; Park and Kim, 2022). Just as an “embodied Internet” proclaimed by Mark Zuckerberg, the metaverse will enable people to have authentic, immersive, and multimodal experiences as if they are in the real world or even more than the real world (Bourlakis et al., 2009; Nevelsteen, 2017; Jovanović and Milosavljević, 2022).

#### Decentralized and editable content

Compared to the former Internet mode where the content was limited to specific groups like the developers, the metaverse entitles every user rights to edit or create content with a virtual nature that involves changing its properties, position, or orientation. Just as conceived by Roblox or Facebook (Jeon, 2021; Zuckerberg, 2021), users can create almost everything they can imagine. Additionally, players are also allowed to co-create or modify others’ shared content (Taylor and Soneji, 2022). More significantly, users can own and run their own digital properties, and the security technologies, such as blockchain, can ensure their personal properties be safe and traceable (Vergne, 2021; Min and Cai, 2022; Vidal-Tomás, 2022).

In light of the above discussion of the metaverse, we, therefore, conceptualize the metaverse as a 3D digital space mixed with the real and virtual worlds, which runs off a lot of limitations (e.g., time, location) of the physical world. It allows users to engage in a variety of activities (e.g., working, learning, training, socialization, transaction) through avatars and interact with the other players and the virtual objects, as well as provides opportunities for users to edit the contents. Users can also enjoy richer, more immersive, and more embodied experiences than ever.

## Definition, framework, and features of the metaverse in education

### Definition of the metaverse in education

As indicated by scholars, education is one of the most significant applications of the metaverse with great potential in the coming future. We believe that the presence of the metaverse can be served as a new educational environment (Suzuki et al., 2020; Prieto et al., 2022; Rospigliosi, 2022); therefore, the metaverse in education can be regarded as an educational environment enhanced by metaverse-related technologies which fuse with the elements of the virtual and the real educational environment. It enables learners to use wearable devices to enter the educational setting without being limited by time and locations and allows them to use digital identities to have real-time interactions with different forms of items (e.g., avatars, intelligent NPCs, or virtual learning resources). As a result, they can feel present as if they are in a real-world educational setting. From this standpoint, it can be seen that applying the metaverse in education can unlock a variety of fantastic learning experiences for learners.

### The framework of the metaverse in education

**Figure 1:**
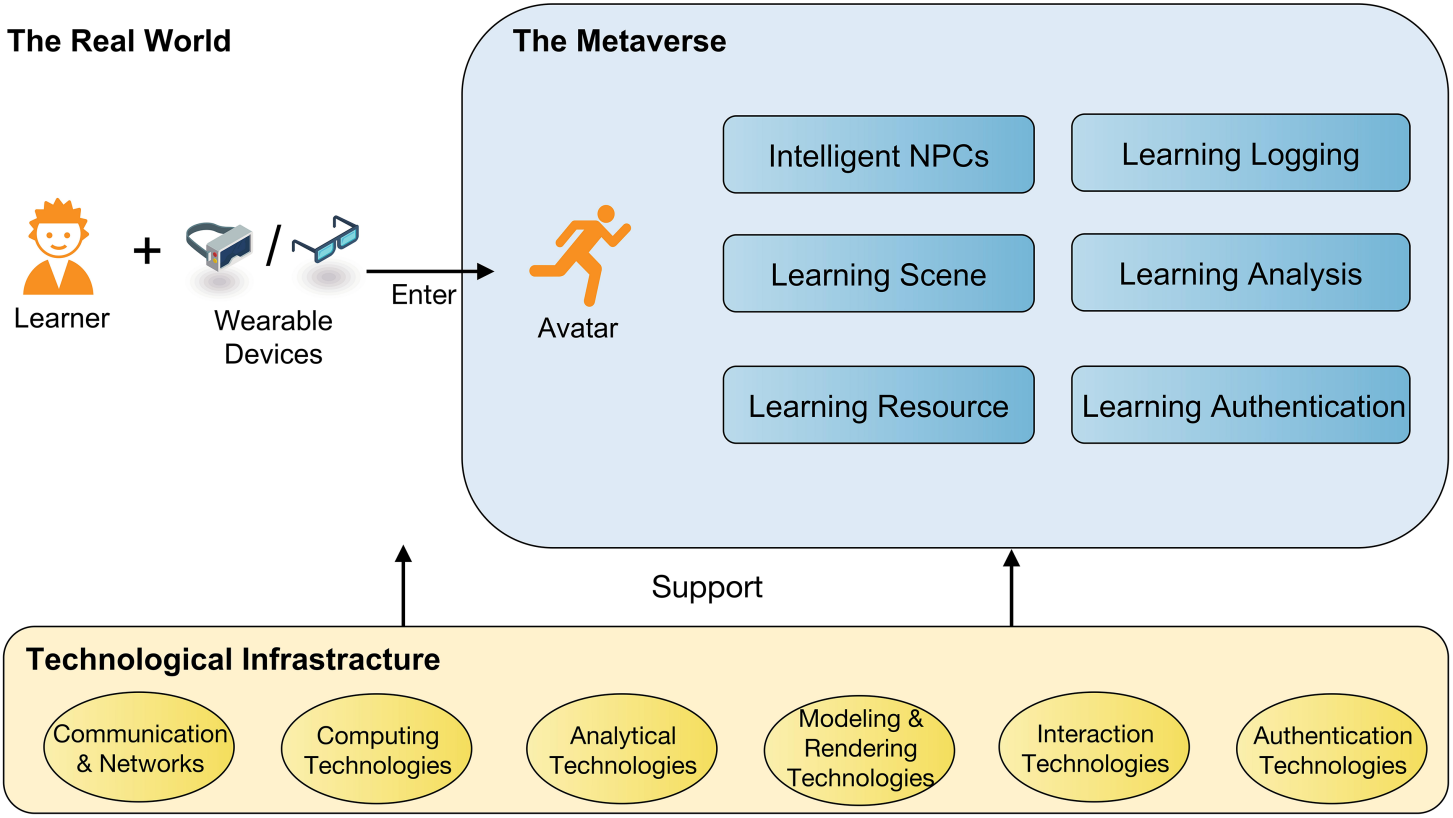
The framework of the metaverse in education.

In previous literature, Kang (2021) proposed a metaverse framework with potential core stacks, including hardware, compute, networking, virtual platforms, interchange tools and standards, payments service, content, service, and assets, as well as introduced its reasons in brief. However, there are no further explanations for the implementation of the metaverse in detail. Park and Kim (2022) divided the metaverse into three essential components (i.e., hardware, software, and contents) and three approaches (i.e., user interaction, implementation, and application) for the metaverse in a general meaning. Some other scarce proposed work of the metaverse in education is from Hwang and Chien (2022). They discussed the roles (i.e., intelligent tutors, intelligent tutees, and intelligent peers) in providing educational services and potential applications of metaverse for educational settings from the perspective of AI. However, the metaverse is not developed by single technologies, such as AI, but integrated with massive technologies. Caring for the few studies that have discussed the metaverse for educational purposes, hence, according to the viewpoints collected from the research papers, we propose a framework for the metaverse in education. As depicted in Figure 1, we shall describe the framework of the metaverse in education and dive into its key components in this section.

At the first sight, the metaverse arises precisely due to the maturity of technologies (Kang, 2021; Yang et al., 2022), that is, the realization of the metaverse in education relies much on up-to-date technologies. Hence, a range of technologies can become the infrastructure of the metaverse in education, which is responsible for providing grand support for the components both in the real world and the metaverse world. Here is a discussion of each component of the technological infrastructure of the metaverse in education.

#### High-speed communication and networks

As indicated by scholars (e.g., Kang, 2021; Yang et al., 2022; Zhang et al., 2022a), wireless communication and high-speed networks, such as 5G or 6G, are the basic requirements for the implementation and work of the metaverse world. With the support of high-speed networks, the metaverse can keep fluency, steadiness, and low latency for data transmission, scene presentation, immediate feedback, and user connection. On another hand, high-speed networks provide learners great opportunities to switch from the physical world to the metaverse educational environments remotely and seamlessly.

#### Computing technologies

Owing to a space gathered by multi-players, the metaverse requires computing technologies (e.g., edge computing, cloud computing, distributed computing) to process, compute, store, transmit, and interchange data and information between the virtual world and the real world, and among users (e.g., Kang, 2021; Zhao et al., 2022). In this case, these technologies can help learners to store, utilize, and share learning data (e.g., learner information, learning records, learning resources) more accurately, efficiently, and synchronically.

#### Analytical technologies

With the rapid development of analytical technologies, related technologies such as AI, big data, and text mining have been deemed useful tools in the educational field (e.g., Park and Jeong, 2022; Yang et al., 2022). As indicated by Hwang and Chien (2022), AI in the metaverse can play an important role in providing intelligent NPC tutors, intelligent NPC tutees, and intelligent NPC peers to support the educational services of arbitration, simulation, and decision-making. Therefore, integrating analytical technologies in the metaverse can help to measure, trace, collect, and analyze the learning data of learners (e.g., learners’ behaviors, emotions, preferences, and performances). Further, in light of these data, the metaverse can not only help teachers to assess learners in a comprehensive way but also provide learners with personalized resources and services.

#### Modeling and rendering technologies

The metaverse aims to create a kind of 3D digital space mixed with the virtual and real world, which includes various simulated or mirrored scenes, avatars, NPCs, etc. At present, there are several modeling and simulation solutions to create virtual items like Sketch Up, Unity, and Blender (Tlili et al., 2022). The global trend of VR or AR research has also made it possible to construct photorealistic 3D content (Wu et al., 2013; Parmaxi, 2020); however, Park and Kim (2022) believed that the metaverse is much more than VR or AR, but a concept closer to XR. Other scholars (e.g., Lv et al., 2022) mentioned technologies like digital twins, holography, and MR (mixed reality) can also be used to model and render the metaverse world. In this sense, modeling and rendering technologies are indispensable to constructing a vivid and colorful educational space with rich details and high fidelity. In addition, they provide great possibilities for some scenes and items in education, which can not be presented in the physical world, to be visualized in the metaverse world.

#### Interaction technologies

Embodied and multimodal interaction is a unique feature of the metaverse compared with the conventional Internet. Interaction technologies like VR, XR, sensors, real-time tracking, IoT, and BCI (brain-computer interface) are necessary for users’ manipulations, navigations, collaborations, and sensory feedback (e.g., vision, audition, and kinesthesia) in the metaverse (e.g., Davis et al., 2009; Genay et al., 2021; Prieto et al., 2022). With the support of interaction technologies, learners can mobilize their bodies to take part in various exploratory learning activities, collaboration, and socialization, to stimulate different sensory organs and get real-time feedback. To some extent, the metaverse can provide learners with authentic and embodied learning experiences.

#### Authentication technologies

Some scholars (e.g., Berg et al., 2019; Vergne, 2021; Thomason, 2022; Yang et al., 2022) stated the most representative authentication technology in the metaverse is blockchain, which can provide transparent, open, decentralized, and reliable services and protect users’ privacy to keep the metaverse world to have a sustainable ecosystem. In this sense, blockchain can not only be used to make learners’ data and works in the metaverse unforgeable and traceable, but also avoid some negative issues, such as fraud or plagiarism.

It should be noted that the technological infrastructure of the metaverse in education and its components are based on the current status of technologies, and it is expected that with the evolution of technology, its components will continue to expand in the years to come. As shown in Figure 1, except for the technological infrastructure, there are still other important components in the real world and the metaverse world need to be taken into account for realizing the metaverse in education.

#### Smart wearable device

Smart wearable devices include headsets or head-mounted displays (HMD), smart glasses, etc., which can be divided into non-see-through, optical-see-through, and video-see-through (e.g., Kang, 2021; Zuckerberg, 2021; Taylor and Soneji, 2022). As stated by Park and Kim (2022), the smart wearable device is a basic hardware component that links the real world and the virtual world. Hence, the smart wearable device can help learners to teleport themselves from the real world into the metaverse and switch between the real and virtual worlds without restrictions.

#### Avatar

In the metaverse, the avatar is the digital representation of the player character (i.e., teachers and learners). The support of real-time tracking technology, recognition technology, or simulated technology has made great improvements in the realism of avatars. Both learners and teachers can customize their avatars, with some features (e.g., dressing style, gender, skin color) similar to or different from themselves. For example, Zhao et al. (2022) mentioned details such as facial expressions and gestures of avatar learners can be captured and lively displayed by scanning users’ physical appearances. Avatars can also be manipulated and shared with the same actions by users with sensors, controllers, or real-time tracking (Genay et al., 2021). Namely, avatars can help learners to express themselves in a new joyful, and completely immersive way, as well as provide them with a sense of being and embodiment to them when they experience the metaverse.

#### Non-player character (NPC)

In the metaverse-based learning environment, there are several special AI-driven roles in the metaverse: intelligent NPC teachers, intelligent NPC learners, and intelligent NPC peers (Huang et al., 2021; Jovanović and Milosavljević, 2022). As indicated by Hwang and Chien (2022), these intelligent agents can play an essential role in supporting arbitration, simulation, and decision-making for educational purposes. It implies that in the metaverse world, learners can get tutoring, seek help, have discussions, or practice skills with NPCs; meanwhile, teachers can also ask for help or simulate teaching with intelligent NPC learners at any time. In this sense, the provision of those intelligent agents can greatly meet personalized needs and enhance interaction for both learners and teachers.

#### Learning scene

In the metaverse, various realistic learning scenes can be simulated and created by modeling and rendering technologies such as digital twins, VR, AR, XR, etc. (Davis et al., 2009; Duan et al., 2021; Lv et al., 2022; Shin, 2022). The scenes can be reproduced like real-world classroom layouts in 3D form or constructed as partially or fully virtual scenes according to the learning contents, especially for that can not be easily seen in the real world, such as universe, marine, forest, historical site, etc. (Wu et al., 2013; Choi and Kim, 2017; Prieto et al., 2022). In addition, the construction of the learning scenes focuses more on the details like texture, color, ornament, etc. (Zhao et al., 2022). For example, Seoul National University (SNU) Bundang Hospital used an XR technology platform to simulate a virtual conference (Koo, 2021). Ko et al. (2022) constructed a virtual classroom with a real classroom layout in the metaverse platform Gathertown.

#### Learning resource

Owing to the modeling and rendering technologies, the resources can be visualized in the metaverse, especially for the invisible or abstract concepts, items, or events in the physical world (Dunleavy et al., 2008; Wu et al., 2013). In addition, rest on the interaction technologies such as VR, AR, XR, or sensors, the learning resources can be presented by multimodal means and allow learners to motivate their bodies partly or fully to interact with them, providing them with real-time feedback and rich sensory experiences (Chen et al., 2011; Myburgh, 2022; Taylor and Soneji, 2022). For example, Yen et al. (2013) introduced AR to visualize the lunar system in teaching astronomy, which allows learners to actively participate in the interaction with the virtual lunar. Moreover, due to the decentralized technologies, the metaverse should allow learners to edit, create and share learning resources (Zhao et al., 2022). For example, the sandbox platform Roblox allows players to create works of a virtual nature, and the works can also be co-created and shared with other players (Jeon, 2021). The virtual social platform ZEPPTO allows users to make AR fashion items, and users can get digital currencies by selling them to other users (NAVER Z Corp, 2022).

#### Learning logging

As the proposed metaverse roadmap stated (Smart et al., 2007), lifelogging, an essential scenario in the metaverse, is the capture, storage, and distribution of daily experiences and information for objects and people. From this point of view, through storage, databases, or tracing technologies, learners’ real-time status information can be presented and shared, meanwhile, learners’ historical information (e.g., footprints, data, assignments, and virtual works) can be recorded and stored in the metaverse. It helps both learners and teachers to review or observe the learning process and conduct some meaningful events (e.g., analyzing behavior or interactive patterns) based on personal experiences (Prieto et al., 2022).

#### Learning analysis

In the metaverse, technologies like computing, databases, or AI play an important role in providing and analyzing huge amounts of data (Yang et al., 2022). The learning analysis module aims to utilize massive data to analyze and display learners’ learning performances and achievements by unit or in all. More significantly, it can make assessing learners’ performance easier, and provide teachers with reliable proof to conduct personalized services for learners. For example, Classting AI is an online class community application that can help to analyze learners’ learning achievements and provide visual and personalized analysis reports (Kye et al., 2021).

#### Learning authentication

The metaverse is a more open, shared, and decentralized digital space than traditional virtual spaces (Hwang and Chien, 2022; Yang et al., 2022). It implies in the metaverse, the storage of learners’ information should be managed and secured as other applications placed nowadays on the cloud with highly secure standards to avoid users’ privacy being violated (e.g., use user authentication and authorization to specify the provided content). In addition, users’ virtual works or digital creations are allowed to be shared with other people, which are expected to be traced and secured. Technologies such as blockchain or NFT (non-fungible token) enable learners’ creations or works to be authenticated and traced which aims to keep the metaverse world safe, persistent, and sustainable (Berg et al., 2019; Vidal-Tomás, 2022).

### Features of the metaverse in education

Based on the proposed framework of the metaverse in education, it can be seen that learning in the metaverse world will not feel the same as in the conventional classroom or screen-based video-conferencing platforms. A comparison of in-person learning, screen-based remote learning, and metaverse-based learning is presented in Table 1. It can be noticed that metaverse-based learning is more than a combination of in-person learning and screen-based remote learning, and it is likely to compensate for the limitations of both. Accordingly, each feature and its significance are interpreted as follows:

**Table 1:** Comparisons of in-person classroom learning, screen-based remote learning, and metaverse-based learning.

Factor	In-person classroom learning	Screen-based remote learning	Metaverse-based learning
The time and location for learners to participate in class	At a fixed time in accordance with the class schedule and school timetable in the real classroom	Available only when a teacher opens a meeting on the video-conferencing platform	Without being limited by either time or location
Learner identity	Real identity	Real identity	Customized and dynamic digital identity (avatar)
The people learners interact with	Real teachers and peers	Real teachers and peers	Real teachers and peers in the form of avatar, or virtual teachers and peers in the form of intelligent NPC
Learning scene	Real learning scenes	Real learning scenes	simulated learning scenes
Learning resource	Mainly printed or multimedia learning resources that learners usually cannot interact with	Mainly multimedia or online learning resources that learners usually cannot interact with	Mainly visualized or decentralized learning resources that allow learners to interact
Learning activity	Primarily based on lectures from teachers Allows learners to participate in a series of learning activities, except in the pandemic era Allow learners to collaborate with peers, except in the pandemic era	Primarily based on lectures from teachers Cannot easily allow learners to participate in some complex learning activities Cannot easily allow learners to collaborate with peers	Primarily a series of contextualized learning activities in 3D learning scenes Allow learners to participate in a series of learning activities virtually, Can support remote collaboration Initiate activities more like inquiry-based or problem-solving tasks Facilitate creative learning activities
Learning experience	Mainly based on face-to-face communication	Mainly based on online communication with video and audio	Mainly based on multi-sensory and embodied participation
Learning objective	Mainly aims to develop low-order cognitions	Mainly aims to develop low-order cognitions	More easily to develop high-order cognitions Mainly aims to achieve more comprehensive learning objectives
Learning assessment	Focus on learning results Based on summative data	Focus on learning results Based on summative data	Combine with formative and summative data Pay more attention to learners’ growth

#### The time and location for learners to participate in class

Conventionally, teachers and learners meet each other in the physical classroom at a fixed time in accordance with the class schedule and school timetable, or it is available only for learners to attend classes when a teacher opens a meeting on the video-conferencing platform (Lee et al., 2022). That is to say, there are limitations of either time or location in classroom learning and screen-based remote learning. As for the metaverse, by utilizing high-speed networks or computing technologies, people can not be constrained by time and location. On one hand, the metaverse can be a near-ubiquitous educational space (Dionisio et al., 2013; Prieto et al., 2022); that is to say, it is always accessible for learners and teachers to enter educational settings through smart wearable devices (Koo, 2021; Kye et al., 2021). Consider a teacher, who is invited to attend a conference out of town and cannot come back to school in class time, he/she can ask their students to use wearable devices to attend classes in the metaverse world, no matter how far apart they actually are. To some extent, dropping the commute will mean less time wasted and more time doing things that matter. On the other hand, technologies such as the high-speed network can help them switch to the real world and the metaverse world fluently and seamlessly, as well as bridge the gap between learning in formal and informal settings (Wu et al., 2013). From this viewpoint, the metaverse allows teachers to innovate the implementation mode of learning: synchronous and asynchronous learning. For example, learners can use avatars to enter the metaverse space and learn by interacting with intelligent NPCs teachers in the metaverse in a predefined way. Therefore, the flexible way of engagement can bring convenience and freedom to teachers and learners. More importantly, it can provide great opportunities for the persistent implementation of education in the post-pandemic era.

#### Learner identity

Whether in the physical classroom or the video-conferencing platform, learners attend classes by their real identities. As for the metaverse, learners can represent themselves in a totally different way. They use their digital identities (i.e., avatars) in customized, realistic, and dynamic forms to attend classes. Avatars are the digital representation of real-world player characters in the metaverse world. When they experience the metaverse, learners can get a sense of being by manipulating and controlling their avatars in a new joyful, and completely immersive way (Park and Kim, 2022; Prieto et al., 2022).

#### The people learners interact with

It is known that learners interact with real teachers and peers in the physical classroom or the video-conferencing platform. Especially on video-conferencing platforms, it is hard for learners to gather and interact with peers and teachers face-to-face through the screen, which leads to some challenges, such as indifference, emotional deficiency, and desocialization (Palvia et al., 2018; Almahasees et al., 2021; Bork-Hüffer et al., 2021; Koo, 2021). Whereas in the metaverse, there are two forms of teachers and peers that learners can interact with: one is avatar teachers and peers, and another is intelligent NPC teachers and peers. On one hand, through interaction with teachers and peers in the form of avatars or intelligent NPCs, learners can get more emotional support and real-time feedback instead of just looking at a grid of faces or boring slides on video-conferencing platforms. On the other hand, intelligent NPC teachers and peers can help to implement learning activities and give personalized support during class or after class. Social constructivism has emphasized that an individual’s knowledge is constructed through social interactions (Pande and Bharathi, 2020); hence, it could be a reason for using the metaverse in education for the cognitive and social development of learners.

#### Learning scene

Conventionally, there are real learning scenes in the in-person classroom and screen-based real learning scenes in the video-conferencing learning platforms. In the COVID-19 era, the construction of learning scenes, such as general scenes (e.g., real classroom) or special scenes (e.g., laboratory), have faced grand challenges (Koo, 2021; Kye et al., 2021). On the contrary, in the metaverse, various learning scenes can be reconstructed virtually based on the real learning environment or simulated in a fully virtual way (Prieto et al., 2022). Take a history lesson on the topic of ancient Rome for instance, it is impossible for people back to ancient Rome in the real world; however, in the metaverse world, the sites of ancient Rome can be reconstructed and represented by technologies. In this sense, it enables learners to experience the learning process in visualized and immersive learning scenes as if they are right there in the real world. Scholars have indicated that when participants attend learning tasks in a simulated and vivid scene, their sense of presence and immersion can be enhanced significantly (Smolentsev et al., 2017; Weech et al., 2019; Maneuvrier et al., 2020).

#### Learning resource

Traditional learning resources generally exist in relatively static forms, such as printed textbooks, printed papers, electronic books, shared courseware, pictures, videos, or other materials (Wu et al., 2013). Learners are less likely to interact with those learning resources. In metaverse-based education, the learning resources are visualized and decentralized which enables learners to interact (Suzuki et al., 2020; Myburgh, 2022). Take a lesson on the topic of the earth, for example, there may be a lecture about the earth with a printed textbook and a demonstration using physical objects of a terrestrial globe and a map in the traditional teaching session; while with the help of AR, the learning resources can be entirely different than before: a 3D spinning earth modeled and augmented by technologies. Learners can observe the virtual earth at 360° by zooming in, zooming out, and rotating it. Hence, in the metaverse, some abstract content can be turned into more concrete by modeling and rendering, thereby enhancing learners’ in-depth understanding. It is also possible for learners to interact with learning resources to facilitate their participation and experience, as well as participate in the process of creating or editing learning resources along with their peers and teachers, which may compensate for certain shortcomings associated with traditional resources.

#### Learning activity

Learning in a physical classroom is primarily based on lectures from teachers and it allows learners to participate in a series of learning activities and collaborate with their peers; however, it is difficult to initiate the above activities in the pandemic era (Almahasees et al., 2021; Ko et al., 2022). Due to some limitations of video-conferencing platforms, screen-based remote learning is mainly lecture-based, but it has little opportunity to initiate some complex learning activities, such as collaboration; as a result, learning in such platforms tends to be passive (Almahasees et al., 2021; Li and Yee, 2022). When it comes to the metaverse, at first glance, the learning activities seem to be contextualized in these vivid and colorful learning scenes, which can greatly enhance their cognitive representation by interacting with virtual objects in 3D perspectives (Dionisio et al., 2013; Myburgh, 2022). As a result of continuous free access and rapid engagement, a further peculiarity of learning activity in the metaverse is that learners can easily collaborate with peers in real-time in virtual forms, such as meetups, conferences, idea sharing, group discussions, presentation panels, or debates, provide learners with more emotional support from peers instead of just staring at a grid of screen (Suzuki et al., 2020; Koo, 2021; Kye et al., 2021; Jovanović and Milosavljević, 2022; Myburgh, 2022; Thomason, 2022). Alternatively, they can schedule appointments to collaborate with partners remotely outside of class. In addition, the digital space with some game-like attributes (e.g., avatar, NPC, or digital items) can be configured to initiate activities more like inquiry-based or problem-solving tasks (Nevelsteen, 2017). Further, the metaverse can offer a decentralized and editable creation space, it can facilitate creative learning activities where learners are able to create virtual works (Choi and Kim, 2017; Ayiter, 2019); meanwhile, it also implies learners can revise and withdraw their actions during the learning procedure aligning with the philosophy of learning from mistakes (Prieto et al., 2022).

#### Learning interaction

In the physical classroom, learners interact primarily *via* face-to-face communication, while on video-conferencing platforms, their interactions tend to be based on video and audio communication (Kye et al., 2021; Li and Yee, 2022). In the metaverse, with the aid of interaction technologies such as sensors, BCI, VR, AR, or XR, learners’ interactions usually involve embodied and multi-sensory participation, as a result, a wide range of learners’ senses (e.g., vision, audition, or kinesthesia) can be greatly stimulated and motivated when they interact in the metaverse (Genay et al., 2021; Zhao et al., 2022). Birchfield et al. (2017) indicated that learning in a multimodal and embodied way can greatly promote learners’ learning interests and performance.

#### Learning objective

Revised Bloom’s taxonomy of learning objectives includes six categories from low to high: remembering, understanding, applying, analyzing, evaluating, and creating (Bloom et al., 1956; Anderson et al., 2001). Due to a few limitations like time, space, or resources, traditional classroom learning or screen-based remote learning primarily concentrates on low-order cognitive development (i.e., remembering, understanding, and applying); however, traditional lecture-based classes make it difficult for students to develop high-order thinking skills (i.e., analyzing, evaluating, and creating) (e.g., Booker, 2007; Arievitch, 2020). Owing to some of its peculiarities, the metaverse enables learners to engage in various types of learning activities (e.g., group work, creative learning, or inquiry-based learning) regardless of whether they are in classes or not, which may help learners to apply, analyze, evaluate or create knowledge more easily throughout the learning process (Prieto et al., 2022; Shin, 2022). Previous studies (e.g., Arievitch, 2020) demonstrated mastery of all levels of learning objectives is based on or merged within the process of complex activities such as problem-solving. As a result, developing learners’ high-order thinking skills can become relatively easier in the metaverse. To some extent, it will generate a series of “ripple” effects on developing learners’ goals from high-order to low-order. In other words, in the metaverse, learners can not only grasp basic knowledge but also develop their skills and competencies for future life to get more comprehensive development during the whole learning process.

#### Learning assessment

In conventional learning environments, teachers often assess learners summatively by learning results (e.g., tests) due to the difficulty of recording learners’ performance and collecting their learning data. (Parmaxi, 2020; Bork-Hüffer et al., 2021; Kye et al., 2021; Jovanović and Milosavljević, 2022; Taylor and Soneji, 2022). In this case, scores will be the only indicator of learners’ learning, resulting in negative effects such as inequality in education. In the metaverse, with the support of learning logging and learning analysis, teachers can assess learners’ performance more comprehensively based on both formative and summative data. More significantly, it emphasizes more on learners’ growth rather than results, thereby breaking free of some limitations of traditional assessment.

## Future potential applications of the metaverse in education

When Mark Zuckerberg presented the rebranding scheme of Facebook in a live-streamed virtual way, it is impressive that some potential applications of the metaverse like gaming, working, or learning were vividly displayed. In the metaverse, both learners and teachers can break free from the restrictions of time and location. More significantly, the peculiarities of the metaverse are going to unlock a lot of amazing learning activities for learners, which enable them to perceive, explore, and create the world in unprecedented ways. Therefore, it can be foreseen that the metaverse world could open a new window for future education. Owing to the features and affordances of the metaverse in education mentioned above, this section will discuss several potential applications for the metaverse in education but is not limited.

### The metaverse assists blended learning

Blended learning, as a learning paradigm, refers to a combination of traditional in-person and online learning (Garrison and Kanuka, 2004; Bonk and Graham, 2006). During the COVID-19 pandemic, screen-based remote learning on video-conferencing platforms such as Zoom, Google Meet, or Teams has become a norm (Almahasees et al., 2021; Kye et al., 2021; Ko et al., 2022). As the pandemic in some regions has leveled off, some schools have begun to ask their learners and teachers back to school, meanwhile, the trend of autonomous learning in remote or blended forms is considered to continue in some lockdown areas. Under the context of the uncertainty of COVID-19, the combination of non-face-to-face and in-person education is still expected to be an available choice for the sustainability of post-pandemic education (Palvia et al., 2018; Bork-Hüffer et al., 2021; Lee et al., 2022; Zancajo et al., 2022). However, scholars have reported several problems linked to learning on video-conferencing platforms, including video-conference fatigue, tiredness, lack of motivation, inability to focus, desocialization, and depersonalization (Almahasees et al., 2021; Li and Yee, 2022).

**Figure 2:**
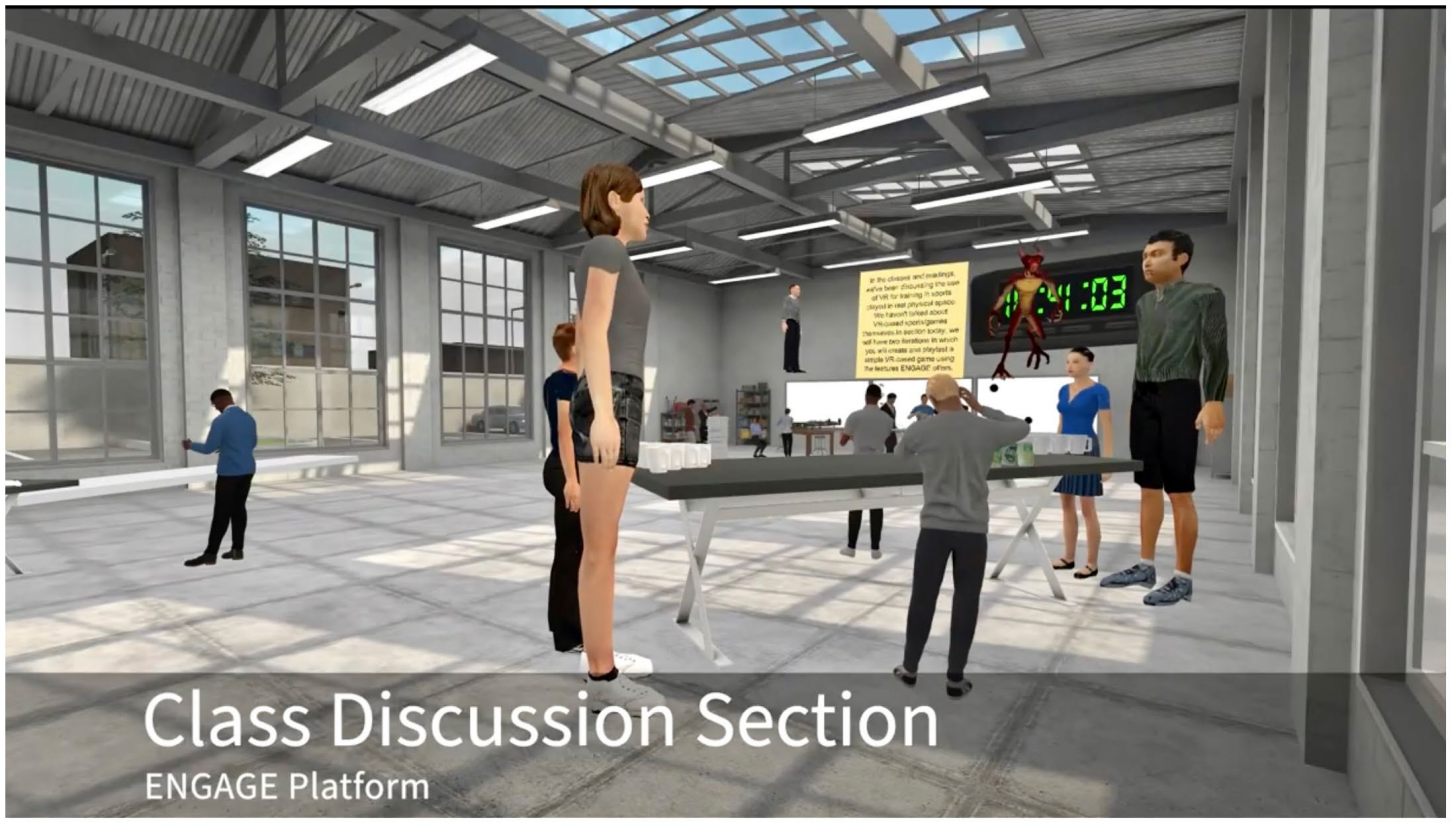
The class discussion section of “Virtual People” in the metaverse (Stanford University, 2021). Reproduced with permission.

For example, in the autumn of 2021, professor Jeremy Bailenson opened a course “Virtual People” (Communication 166/266 Syllabus) in the form of blended learning at Stanford University (2021). 263 students with VR headsets gathered in the metaverse by using Zoom and ENGAGE platforms. Participants were asked to finish the reading task before class. During the metaverse learning sessions, learners needed to engage in a variety of activities virtually and remotely, such as large group field trips, small group panels, quizzes, and creating virtual spaces both alone or together. Figure 2 shows the picture of the class discussion section of “Virtual People” in the metaverse platform.

In this sense, the metaverse enables both teachers and learners located in different physical places (e.g., at home, in lockdown areas, overseas), to have great opportunities to involve in educational settings through wearable devices. From this standpoint, simultaneous or asynchronous in-person and remote learning can be easily realized in the metaverse. Learners can use avatars to participate in various learning activities (e.g., lecture, individual work, group panel, collaborative work) and interact positively with either real or virtual teachers and peers in various learning scenes. When they are in the metaverse, they will feel as if they are right in the same space together, with more incredible experiences and peer support than not just looking at a grid of faces or boring slides on the screen. Through such forms of learning, the participation and learning interest of learners might also be greatly enhanced. More significantly, the problems existing in current video-conference learning may have a practical way to be addressed. Given this trend, the metaverse can develop to evolve multiple new paradigms of blended learning for years to come to facilitate better learning engagement and experience for learners (Ko et al., 2022).

### The metaverse assists virtual experiment learning

Virtual experiment learning plays an essential role in natural science (physical science and life science) curricula (Vergne, 2021). As indicated by some scholars (Gamage et al., 2020; Zhang et al., 2022b), virtual experiment learning faces many challenges, including limited funding for materials and infrastructures, a lack of solutions to the closure of physical laboratories due to COVID-19, etc., which led to practical experiment training being less prioritized than theoretical learning (Myburgh, 2022).

With the assistance of modeling and rendering technologies, a virtual laboratory can be recreated virtually in the metaverse, as can the experiment apparatuses projected in 3D to the virtual world to practice a variety of virtual experiments. Meanwhile, learners are allowed to participate in various virtual experiments with real-time interactions through interaction technologies. Considering the features of the metaverse in education, the following potential applications of the metaverse to virtual experiment learning are listed, with references to several position papers (Wu et al., 2013; Gamage et al., 2020; Vergne, 2021; Myburgh, 2022):

1. To assist the experiments that could be risky, irreversible, or toxic in the real world, e.g., an experiment with a potential risk of explosion;
2. To assist the experiment conditions and scientific phenomena that could not be possible in the real world, e.g., an experiment that needs to be carried out in a vacuum;
3. To assist the experiments that need relatively high costs and funds in the real world, e.g., an experiment that needs expensive equipment and materials;
4. To assist the experiments that react slowly or need long-term observations and records in the real world, e.g., an experiment needs learners to observe and record the whole growth stage of an insect.

In this sense, applying the metaverse for virtual experiment learning can break through restrictions in the physical world, such as space, funds, sites, equipment, or potential risk, as well as make learners possible to observe, measure, record, and manipulate experiments in stand-alone or collaborate way remotely. Further, it can help to acquire skills through continuous practice as well as promote learning from mistakes. Based on these merits, applying the metaverse in virtual experiment learning could be promising.

### The metaverse assists language learning

Since the 21st century, language learning has been fundamental for acquiring bilingual or multilingual proficiency for K-12, higher education, or professional field (Peters and Fernández, 2013). However, for several reasons, such as lack of contextual practice or interaction, traditional language learning has been taken passively either in the classroom or out of class (Liang et al., 2021). As indicated by scholars (Parmaxi, 2020; Park, 2021; Guo and Gao, 2022; Lee and Jeong, 2022; Ryu, 2022), the emergence of the metaverse can show great potential to promote language learning.

There are several reasons for adopting the metaverse in language learning. At first, language learning requires a strict learning context, especially for listening and speaking classes (Chen et al., 2021; Lin and Wang, 2021). For example, the objective of one speaking lesson is to grasp spoken skills by practicing the dialogue on the topic of asking for flight information in an airport context. For example, in the real world, it is quite unrealistic for teachers to take a whole class of learners to the airport or invite the airport staff to come to the school. However, in the metaverse, learners can attend various learning activities, such as role-plays and dialogue practice, with avatar partners or with a predefined intelligent NPC airport staff or air hostess, in a simulated airport setting. In this case, the metaverse could situate language learners in a simulated and vivid learning context, which allows them to experience an immersive language learning process and develop their language skills with teachers and peers. Besides, language learning requires constant and long-term practice after class. For example, considering an EFL (English as a foreign language) learner who is on summer vacation, it is quite difficult for his/her to call friends to gather together to practice English (e.g., listening, translating, speaking). While in the metaverse, this learner could invite his/her partner to walk into the virtual world by avatar, and then practice dialogues in a former-created scene or a recreated scene remotely. If a partner is unavailable for some reason, from the perspective of intelligent agents, it is helpful for learners to the provisions of intelligent language peers who are capable of language practice. In this case, the metaverse can provide language learners an appropriate space to have real-time practice and interaction with real or NPC roles freely out of class, which may also help their language ability transfer to the real context effectively.

### The metaverse assists competence-based education

Competence-based education (CBE) is a leading paradigm for educational reform in the Vocational Education and Training (VET) sector, in which the competences (e.g., knowledge and skills) needed in later vocational practice form the foundation for curriculum development rather than the general academic subjects (Koenen et al., 2015; Wijnia et al., 2016; Antonietti et al., 2022). In most cases, VET is built upon the alternation between theory and practice. However, due to the COVID-19 pandemic, how to conduct CBE is going to be a thorny problem (Liang et al., 2022).

Take the SNU Bundang Hospital as an example, a virtual conference was simulated by an XR technology platform, which allows participants in different locations to wear a 3D headset to attend a training course and used avatars to have active discussions with others. At the same time, with the help of VR, a high-resolution camera, and fluorescent imaging equipment, a lung cancer surgery was conducted in a smart operating room of SNU Bundang Hospital and the real-time surgery scene with high-resolution was displayed on the conference screen. As participants indicated, they were observing the surgery procedure as vividly as they were in the real operating room (Koo, 2021).

In this sense, the metaverse is expected to offer a potential solution for CBE. On the one hand, it enables both teachers and learners to switch between the classroom scenes and the professional scenes seamlessly and helps the learners acquire general and professional knowledge by observing the process in a virtual environment remotely; on the other hand, it can constantly situate learners in a professional training environment to practice skills with target groups whenever and wherever possible instead of going to the practice base with time and fare waste. Consider a learner, who is a preservice nurse, he/she might need to practice his/her first aid or nursing skillsets, and it is almost impossible to find patients to do this in the real world. In this case, it is quite helpful for him/her to have a group of intelligent NPC patients in simulated hospital or clinic scenes in the metaverse.

### The metaverse assists inclusive education

Inclusive education is introduced to enable every child to receive education and necessary support in mainstream school settings, regardless of their special needs (Spandagou, 2021; Sahli Lozano et al., 2022). Children with special needs mainly refer to the group of disabilities; also, the spectrum is extended to include children who are abandoned, abused, or mentally ill (Arora and Sahu, 2015). However, it is challenging for most educators to get students with special needs to be accepted by general students and learn together without barriers. Based on the peculiarities of the metaverse, it is explicable that the metaverse could be served as an ideal zone that allows children with special needs to have the possibility to study with general learners together (Duan et al., 2021).

At first, the identity difference is an important reason that hinders the integration of those learners with special needs in general schools with general learners (de Boer et al., 2011; Schwab, 2017). The digital identities in the metaverse can rebuild those learners’ images to eliminate special identity labels and discrimination, which makes it possible to help them engage in the learning activities with general students with confidence and a sense of belonging. Next, as part of or whole sensory or cognitive obstacles, the metaverse technologies (e.g., AI, sensors, or BCI) can extend those learners’ affordances of organs and senses to communicate and interact with the general learners normally and obtain sensory stimuli and cognitive development during the learning process. Moreover, owing to the difference in learning between learners with special needs and general learners, the metaverse can assist learners with personalized learning and special services according to the physical and emotional data *via* the technologies of computing, big data, learning tracks, and so on. Accordingly, the metaverse can meet with the philosophy of inclusive education that recognizes and appreciates the diversity of every learner; more significantly, it provides all learners an equal chance to involve in mainstream school.

## Challenges of the metaverse in education

Although the metaverse provides us with innovative perspectives for education, we should be vigilant about a series of challenges of the metaverse for either educational or other purposes. In this section, we will mainly discuss four challenges of the metaverse in education.

### Technology and equipment

A well-designed and affordable smart wearable device is essential for both learners and teachers to teleport to the metaverse world (Parmaxi, 2020). As Park and Kim (2022) indicated, the hardware (e.g., HMD) is now quickly enhanced as a result of technological advancement, but it still needs further improvement. For example, it is being reported (Weech et al., 2019; Tlili et al., 2022; Xi et al., 2022) that users could get some symptoms (e.g., cybersickness, blurred vision, or dizziness) or even fall into the ground after putting on the wearable devices for a period of time, which may bring about a potential security risk in practice. Also, price is an important factor of hardware, but the current cost of equipment is still too high for most people (Taylor and Soneji, 2022). For the user interface, it is worthwhile to think about how to meet the needs of free access, high fidelity, visualization, immersion, or multi-sensory interaction of the metaverse. The current hardware or software is far from the technical standard for the prevalence of applying the metaverse in the educational field. From an evolutionary point of view, the fast-growth technologies of 5G networks, VR, or digital twins are showing promise for the metaverse. Hence, it is necessary for technology companies and departments concerned to develop more advanced solutions for adopting the metaverse in education.

### Privacy and data security

It is a critical issue that the users’ privacy and data security whether on the 2D Internet or in the 3D virtual world. In the metaverse, data is the basic form of governance, which allows more detailed data from users to be collected (Kye et al., 2021; Lv et al., 2022; Zhao et al., 2022), such as facial images, physical state (heart rate, blood pressure, disease, etc.), transactions, consumption records, etc. In addition, it is more easily for learners with little social experience to be exposed to criminal events (e.g., fraud, surveillance, leakage) due to a higher level of online anonymity in the metaverse. Once it happens, it will violate the learners’ privacy, and even seriously affect their normal life. Moreover, the works and creations of both teachers and learners may also have the potential risk to be plagiarized. In this case, the related rules and regulations (e.g., real-name authentication) are expected to be enacted and regulators that play the same role as police in the real world are urgently needed; also, it is necessary to make the works and creations in the metaverse traceable with the help of the technologies such as cryptocurrency, NFT and blockchain (Berg et al., 2019; Thomason, 2022; Vidal-Tomás, 2022). Otherwise, the metaverse will be a lawless digital space.

### Ethics and morality

Due to the high degree of freedom, users in every corner of the world can have access to the metaverse (Kye et al., 2021). There are new concerns (e.g., the different ideologies and worldviews, simulated experiments, data stealing, racial problems, religious conflicts, bullying, violence, etc.) that may potentially cause cross-national, cross-racial, cross-religious, or cross-gender ethical challenges, by the appearance of virtual “I” (Dionisio et al., 2013; Park and Kim, 2022). In addition, Therefore, it is an urgent problem how to establish a well-organized metaverse with rules and ecosystems. At the same time, cultivating learners’ citizenship in the metaverse *via* ethics and legal education will also be essential.

### Addiction

The high immersion and presence close to the reality created by the sensor and virtual technologies, and plenty of scenarios and items that exist in the metaverse but miss in the real world, make learners more easily indulge in such a bizarre metaverse world (Choi and Kim, 2017; Weech et al., 2019; Kye et al., 2021; Prieto et al., 2022). However, it is considered to be an inevitable risk that young learners, who lack self-discipline and self-control, may fall into a state of addiction, which may lead to potential damage to their physical and mental health (Xi et al., 2022). Therefore, relative guidance from both teachers and parents will be required for learners to balance their time in and out of the metaverse world and avoid excessive lingering on the metaverse to prevent false spiritual satisfaction from the technologies.

### Identity and social interaction

In the metaverse, digital identities can directly reflect users’ egos to participate in various types of activities (Davis et al., 2009). As the boundary between the real world and the virtual world gets blended, users may be bewildered by their “real-me identity” and “virtual-me identity” (Kye et al., 2021; Xi et al., 2022). Additionally, if the learners rely much on the social connection established between avatars and NPCs in the virtual world, over time, learners will gradually have emotional and social barriers, making it difficult to establish the social relationship in the real world; at the same time, there is also a possibility that NPC teachers may be a threat for the status of real teachers. Hence, a timely guide to learners, from society, school, and family, for recognizing the difference between reality and the virtual world, rationally treating the metaverse, and paying attention to real-world interaction, should be necessary.

## Future research topics of the metaverse in education

As stated in the above sections, the metaverse could play a significant role in education. The evolution of emerging technologies can provide various opportunities for applying the metaverse in education. However, limited studies focus on the metaverse in education at present. We believe that the number of articles about this field will grow rapidly in the coming years. Accordingly, for the extension of future research, a range of potential research issues of the metaverse in education, are covered as follows:

1. Designing the metaverse models or frameworks for educational purposes. So far, the metaverse is under construction (Prieto et al., 2022), and it requires infrastructure to be of a high standard adapted to common practices. The designs and frameworks of the metaverse including both hardware and software, are the foundation for educational practices. It is considered that multiple factors should be involved in the metaverse design with regard to school administrators, teachers, and learners, such as accessibility, safety, humanity, trust, educational capabilities, and learners’ cognitive characteristics. Besides, more effort should be paid into peculiar and additional design features for education. For example, a specific environment might offer the ability to “airdrop class notes” from one learner avatar to another learner avatar.
2. Enacting the metaverse rules and principles in education. Although the metaverse is a possible digital space for education with rich boons, there are still potential challenges of privacy, security, and ethics raised in the fifth section. Learners, especially teenagers, are in a critical period of physical and mental development. Current issues in learning activities may have a profound impact on their future life. Therefore, establishing and employing strict rules in metaverse-based educational settings should be urgently needed.
3. Investigating attitudes of school administrators, teachers, and parents towards adopting the metaverse for educational purposes. It can be foreseen that applying the metaverse can provide not only great opportunities but challenges for teachers and school administrators. In addition, the metaverse can change the way how learners study both in school and at home. Therefore, it is worth investigating the attitudes of school administrators, teachers, and parents towards employing the metaverse for educational purposes, which is expected to provide valuable references for future design, administration, and educational practice of the metaverse.
4. Teachers’ professional development in relation to the metaverse. It is universally believed that teachers play a fundamental role in successful education and bringing about educational reform. As an emerging educational technology, the metaverse could provide various opportunities for teachers. To this end, how to make a good preparation for teachers to teach by adopting the metaverse is a complex and multitudinous undertaking (Belei et al., 2011; Lee and Jeong, 2022). Moreover, the presence of the metaverse also brings about new appearances for teacher education held in a brand new virtual space. Consequently, teacher education and professional development may become indispensable issues in educational research about the metaverse.
5. Exploring the cognitive and non-cognitive impact on learning of learners with the metaverse. This could be a promising direction for educational researchers to involve. As the educational implementation and paradigm in relation to the metaverse might be much different from the current education, it is needed to conduct exploratory research to compare different academic performances of learners varied in grades and ages by the metaverse and conventional technology. At the same time, under such an innovative environment with high immersion, presence, and freedom, it is also worthwhile to investigate the effect on learners’ both cognitive factors (e.g., attention, memory) and non-cognitive factors (e.g., learning attitude, learning motivation) with the metaverse. Moreover, it is helpful for educators to have an in-depth understanding of the learners’ behaviors in the smart environment fused with the virtual world and the real world by observation and analysis, so as to be able to know the social impact of the metaverse and help develop more effective learning strategies for learners.
6. Comparing the learning and teaching effectiveness among the metaverse and other learning environments as well as among the different metaverse platforms. When applying new technology for educational purposes, it is crucial to conduct comparative research to find out the relatively effective educational environments for teaching and learning. For example, will learners perform better in the metaverse world compared to in-person learning in the physical classroom or screen-based remote learning? Will learners have the same perceptions and performance in different metaverse platforms? Compared to the different environments, which part of the performance of learners will be significantly enhanced? We believe these mentioned topics deserve to be explored.
7. Proposing new thoughts on the methodological and pedagogical model in line with the metaverse. Due to its peculiarities, the metaverse can be seen as an ideal space for future education, where the conventional pedagogical model will change from static to dynamic represented, and learners are gradually the center of the teaching-learning process (Wu et al., 2013). In this sense, the paradigm of conventional education will be broken. With this in mind, it is important to explore the new methodological and pedagogical models that can fit into the metaverse world.
8. Discussing the existing pedagogical theories for metaverse-based education. As a new concept, the metaverse in education will raise new discussions on pedagogics. It is essential to reconsider and revise the existing technology-enhanced pedagogy. Moreover, based on the new features of the metaverse, researchers are expected to propose new thoughts on pedagogical theories for the use of the metaverse based on those theories, like embodied cognition, situated cognition, extended cognition, distributed cognition, flow theory, cognitive load theory, and the technology acceptance model.
9. Developing an educational assessment framework based on the metaverse or employing the metaverse as an assessment approach. As reported by scholars (Parmaxi, 2020; Bork-Hüffer et al., 2021; Kye et al., 2021; Jovanović and Milosavljević, 2022), wherever in either traditional or brand-new educational settings, it is a hard task for teachers to observe learners’ performance and collect learning data during the process; therefore, much attention has been paid to learners’ learning results rather than their learning performance during the process. In the metaverse, with the aid of AI, computing, storage, etc., learners’ performance can be recorded and analyzed accurately during the process. Therefore, various forms of assessment results can be produced, for example, a learning analytical report with both formative and summative data. This indicates the metaverse can provide an alternative way of assessment in a systematic unbiased way. From this perspective, a well-organized assessment framework should be developed, in that some indicators need to be added or adjusted with the application of the metaverse.
10. Uncovering innovative applications and case studies in different disciplines and domains in the metaverse. In the fourth section, we have discussed some potential applications of the metaverse in education (i.e., blended learning, competence-based education, inclusive education, and virtual experiment learning). It can be seen that, in the metaverse, learning in some disciplines and domains will be much different than before, such as physics, chemicals, geography, EFL, medical and nursing education, or communication studies. Hence, it is suggested that researchers could extend their studies to uncover the metaverse applications in different educational domains and provide design cases.

## Conclusion

The technical leaps of high-speed communication, computing, AI, and virtual technologies have offered great possibilities for developing the metaverse (Park and Kim, 2022; Thomason, 2022). As Gartner (2022) predicted, nearly 30% of people will spend 2 h a day in the metaverse for work, entertainment, education, and socialization by 2027. In terms of education, the presence of the metaverse is a brand new concept compared to existing educational technologies. As discussed above, the metaverse can bring about great opportunities and innovations for education. To some extent, a variety of obstacles and limitations in current education could be broken through in the metaverse world. More significantly, the continuous concern on the metaverse even indicates the trend and direction of future education (Park and Jeong, 2022). Accordingly, it can be predicted that more and more educational researchers will actively engage in studies of the metaverse in education in the near future.

In addition, it should be noted that introducing the metaverse into the educational field may trigger several controversial issues (e.g., security, ethics, or addiction) that deserve further discussion; otherwise, the “metaverse” will be a “metaworse.” As for educational researchers, it is more significant to ponder over how to take advantage of the metaverse to overcome the limitations of current education and maximize its positive effects on future education. Hence, as for education, the arrival of the metaverse is thought-provoking and eagerly expected.

## Author contributions

XZ and YC: conceptualization and methodology. YC: writing—the original and final manuscript writing. XZ, LH, and YW: writing—review and editing. XZ and LH: supervision. All authors contributed to the manuscript and approved the final version of the manuscript.

## Funding

This work was financially supported by the National Humanities and Social Sciences Scientific Research Program of the Ministry of Education in China (grant number: 21YJA880027), Wenzhou City Philosophy and Social Science Fund in China (grant number: 22wsk669), and the Graduate Scientific Research Foundation of Wenzhou University in China (grant number: 316202101015).

## Conflict of interest

The authors declare that the research was conducted in the absence of any commercial or financial relationships that could be construed as a potential conflict of interest.

## Publisher’s note

All claims expressed in this article are solely those of the authors and do not necessarily represent those of their affiliated organizations, or those of the publisher, the editors and the reviewers. Any product that may be evaluated in this article, or claim that may be made by its manufacturer, is not guaranteed or endorsed by the publisher.
